# Supplementary material for: Admission criteria in critically ill COVID-19 patients: A physiology-based approach
Source: PLoS One. 2021 Nov 29;16(11):e0260318. doi: 10.1371/journal.pone.0260318 (PMC8629252; doi:10.1371/journal.pone.0260318)
Supplement: S1 Table — (DOCX) [file pone.0260318.s001.docx]

**Supporting information**

**S1 Table. Baseline characteristics between all ICU patients and patients admitted in ICU from Intensivist consultation.**

|  |  | **Total ICU** |  | **ICU admitted** | **p value** |
| --- | --- | --- | --- | --- | --- |
| **DEMOGRAPHIC DATA** | | | | |  |
| Number | n | 81 |  | 63 |  |
| Age | years | 68 ± 11 (29–89) |  | 69 ± 9 (38-89) | 0.67 |
| Male | n (%) | 65 (79) |  | 51 (81) | 0.87 |
| BMI | kg/m^2^ | 28.0 (25.2-32) |  | 28 (24.6-32.3) | 0.60 |
| SAPS |  | 47 ± 17 (13-94) |  | 42 (33-56) | 0.13 |
| NEMS |  | 34 ± 9 (9-49) |  | 31 ± 10 (18-39) | 0.004* |
| **COMORBIDITIES** | | | | |  |
| Arterial Hypertension | n (%) | 48 (59) |  | 42 (67) | 0.22 |
| Ischemic cardiopathy | n (%) | 20 (24) |  | 18 (29) | 0.54 |
| Diabetes | n (%) | 30 (37) |  | 23 (37) | 0.97 |
| OSAS | n (%) | 10 (12) |  | 10 (16) | 0.60 |
| COPD | n (%) | 12 (15) |  | 9 (14) | 0.86 |
| **HEMODYNAMICS** | | | | |  |
| Systolic arterial pressure | mmHg | 129 (120–140) |  | 127 (115-140) | 0.47 |
| Diastolic arterial pressure | mmHg | 65 (60–75) |  | 65 (60-72) | 0.93 |
| Heart Rate | bpm | 85 (76–96) |  | 87 (77-100) | 0.57 |
| Temperature | °C | 37.0 ± 0.9 (35.8-39) |  | 36.8 (36.2-37.9) | 0.33 |
| Lactate | mmol/L | 1.2 (0.8–1.6) |  | 1.6 ± 1.1 (0.5 – 6.9) | 0.31 |
| **RESPIRATORY** | | | | |  |
| SpO_2_ | % | 92 (88-96) |  | 88 (55-100) | 0.31 |
| paO_2_ | mmHg | 65 (52-87) |  | 58 (49-81) | 0.15 |
| paCO_2_ | mmHg | 36 (32-42) |  | 35 (32-44) | 0.34 |
| **LABORATORY** | | | | |  |
| ASAT | U/L | 47 (36-85) |  | 49 (44-85) | 0.72 |
| ALAT | U/L | 33 (21–49) |  | 38 (33-54) | 0.38 |
| Leucocyte | G/L | 7.8 ± 4.7 (2.0-35.0) |  | 9.1 ± 2.0 (2.3-12.3) | 0.12 |
| Lymphocyte | G/L | 0.7 (0.5–1.0) |  | 0.6 (0.4-0.6) | 0.23 |
| C-Reactive-Protein | mg/L | 189 ± 110 (6-534) |  | 135 (116-237) | 0.006* |
| Ferritin | ng/mL | 2379 (864–3234) |  | 1781(1308-4320) | 0.85 |
| LDH | U/L | 582 (400–720) |  | 598 ± 213 (416-1048) | 0.80 |
| Creatinine | μmol/L | 317 ± 96 (10–574) |  | 110 ± 9 (50-410) | 0.52 |
| Creatinine Kinase | U/L | 235 (101–367) |  | 267 (172-573) | 0.19 |
| Platelets | G/L | 197 ± 86 (82-458) |  | 198 (150-254) | 0.05 |
| Bilirubin total | μmol/L | 8.5 (6.5–11.9) |  | 9.1 (7.1-16.5) | 0.67 |

To determine whether the *ICU-admitted* group was a representative sample of the entire ICU patient group, an extra-comparison between the *ICU-admitted* group (patients admitted in ICU exclusively from the intensivist consultation) and the *whole-ICU* population (all ICU patients admitted from intensivist consultation, from other hospitals and from the emergency department, ED) were further performed. Data comparison between the *whole-ICU* group and the *ICU-admitted* group*,* concerning clinical and biological data. Continuous measurements are presented as mean ± SD (min-max) otherwise as median (25^th^-75^th^ interquartile) if they are not normally distributed. Categorical variables are reported as counts and percentages.
